# Dynamic alterations in stool microbiota composition following faecal transplantation in patients with inflammatory bowel disease

**DOI:** 10.1002/ctm2.70888

**Published:** 2026-11-05

**Authors:** Evgenii I. Olekhnovich, Tatyana N. Kalachnyuk, Elena S. Zhgun, Artem B. Ivanov, Dmitry E. Fedorov, Ksenia M. Klimina, Vladimir A. Veselovsky, Daria I. Boldyreva, Andrey K. Larin, Roman I. Matorin, Svetlana V. Lyamina, Dmitriy N. Andreev, Igor V. Maev, Irina Balazs, Vadim M. Govorun, Alexander V. Pavlenko, Julia A. Bespyatykh, Elena N. Ilina

**Affiliations:** ^1^ Lopukhin Federal Research and Clinical Centre of Physical‐Chemical Medicine of the Federal Medical Biological Agency Moscow Russia; ^2^ ITMO University Saint Petersburg Russia; ^3^ A.I. Evdokimov Moscow State University of Medicine and Dentistry, Ministry of Healthcare of the Russian Federation Moscow Russia; ^4^ Research Institute for Systems Biology and Medicine Moscow Russia; ^5^ Center for Bio‐ and Medical Technologies Moscow Russia

Dear Editor,

Faecal microbiota transplantation (FMT) is being investigated for its impact on gut microbiota in various diseases, including inflammatory bowel disease (IBD). Further data are needed on FMT effectiveness across IBD subtypes and optimal therapeutic regimens. New analytical methods can improve understanding of IBD pathogenesis and FMT effects, and facilitate standardised guidelines for donor screening, FMT prescription, and success evaluation. In this exploratory single‐arm longitudinal study, FMT effects on gut microbiota in IBD patients were investigated over one month. Compositional and trajectory analyses quantified microbiome restructuring and shifts towards donor and healthy profiles, accounting for concurrent reduction in disease activity. Donor‐derived colonisation was assessed, and taxonomic and functional changes were characterised using differential ranking and pathway enrichment. Given the small sample size (*n* = 14), the study was not powered for confirmatory between‐group comparisons; between‐group results are exploratory.

Fourteen patients (eight Crohn's disease [CD] and six ulcerative colitis [UC]) with active disease (HBI 5.4 ± 0.8; SCCAI 6.7 ± 0.8) were enrolled and underwent endoscopic FMT (100 mL donor faecal suspension into the jejunum) alongside standard anti‐inflammatory therapy (Table 1 and Figure 1; Supporting Information File S1). Stool samples were collected before FMT and at 2, 7, 14 and 30 days after FMT and underwent shotgun metagenomic sequencing (Supporting Information File S2). Six healthy donors screened according to the previously published algorithm^1^ provided faecal material. Taxonomic and functional profiles were generated for the study cohort (Supporting Information Files S3 and S4), and an external reference cohort was used for taxonomic comparison (Supporting Information File S5).^2^ Multivariate analysis comprised non‐metric MDS based on Aitchison distance, PERMANOVA, and multiple regression on distance matrices (MRM), while robust Aitchison PCA (RPCA) was performed on merged data with an external reference cohort; Axis 1 served as a quantitative phenotype for trajectory comparison. Differential rankings and log ratios were complemented by gene set enrichment analysis, and donor‐derived microbes were detected in post‐FMT recipient metagenomes (Supporting Information File S6).^3^ Longitudinal analyses used mixed and generalised linear modelling, with post‐hoc power analysis.

**TABLE 1:** Patients’ baseline characteristics.

Parameter	Crohn's disease (CD) (*n* = 8)	Ulcerative colitis (UC) (*n* = 6)
Gender	7 M; 1 F	3 M; 3 F
Age	41.9 ± 12.0	30.8 ± 14.2
Age at manifestation	37.9 ± 11.0	29.2 ± 14.5
CD local	7 L1; 1 L3	–
CD phenotype	6 B1; 2 B2	–
UC disease extension	–	2 E1; 4 E3
Complications	Three arthritis and two gut resection (one in the small intestine and one in the rectum)	One arthritis
Medication intake in the last 6 months	5‐ASA (*n* = 3), Antimicrobials (*n* = 3), Antidepressants (*n* = 1), Probiotics (*n* = 1), Glucocorticoids (*n* = 1)	5‐ASA (*n* = 4); 5‐ASA + Glucocorticoids (*n* = 1)
Basic therapy	5‐ASA (*n* = 4); 5‐ASA + Glucocorticoids (*n* = 4)	5‐ASA (*n* = 6)
Clinical response ratio	7/8 (∼88%)	5/6 (∼83%)

Data are presented as mean ± SD unless stated otherwise.

Abbreviations: CD, Crohn's disease; UC, ulcerative colitis; M, male; F, female; 5‐ASA, 5‐aminosalicylic acid.

**FIGURE 1:**
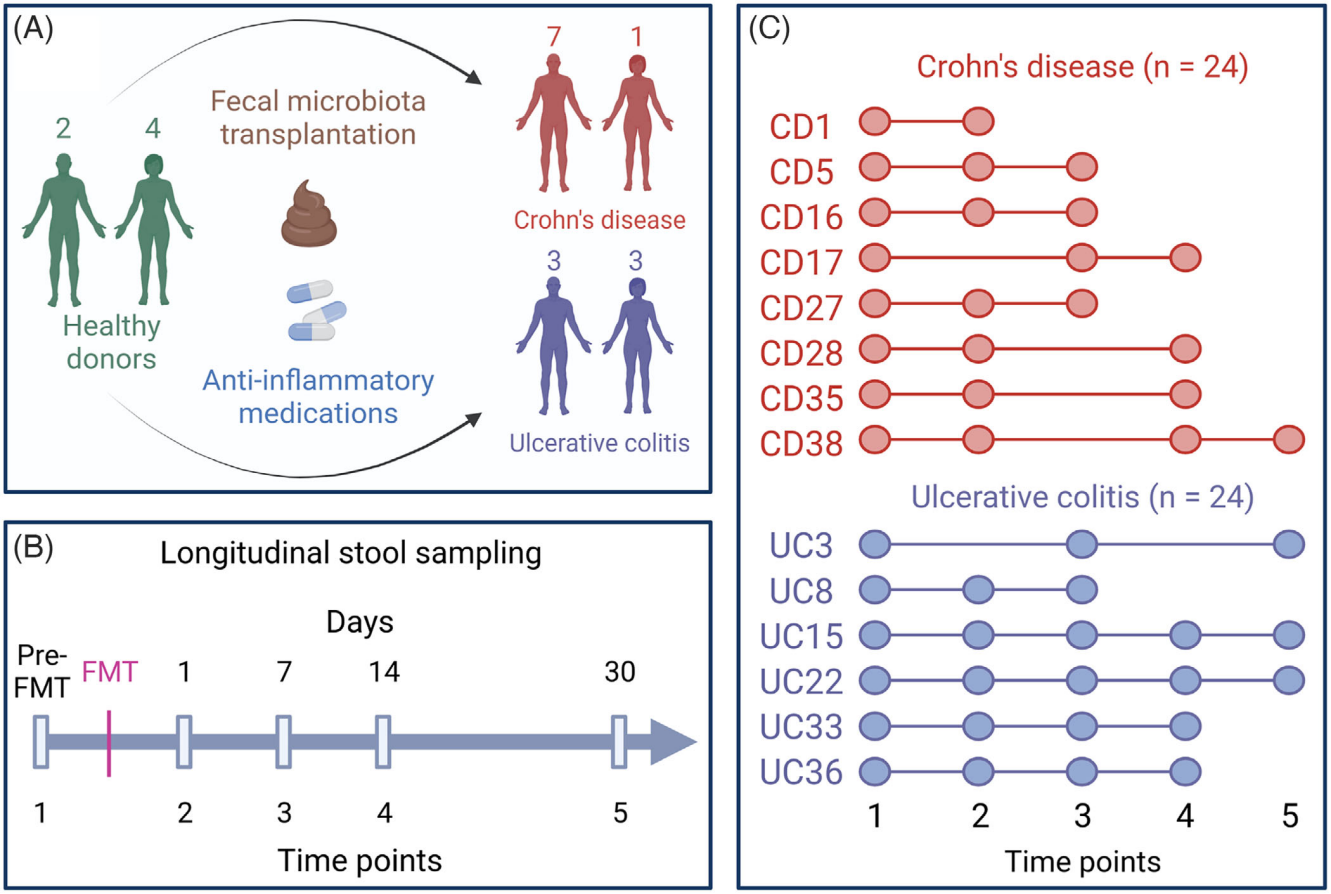
Study design (Created with BioRender.com). (A) Schematic representation of the patient treatment scheme; colours denote disease groups. (B) Longitudinal stool sampling scheme; the magenta marker indicates the time point of the FMT procedure. (C) Stool sampling time points for each patient, with patient IDs shown on the left; colours denote disease groups. FMT, faecal microbiota transplantation.

Within‐subject effects were first assessed using PERMANOVA with patient stratification, which confirmed that both clinical index and time point significantly structured microbial composition (*R* ^2^ = 0.046, *p* = 0.018; *R* ^2^ = 0.039, *p* = 0.002, respectively), indicating progressive within‐subject restructuring over the follow‐up period. Between‐subject effects were then tested using MRM, which showed that patient identity strongly predicted microbial composition (coefficient = −1.53, *p* = 0.0001), while donor identity did not (coefficient = −0.078, *p* = 0.64) and disease was borderline (coefficient = −0.095, *p* = 0.061), indicating donor identity explained little variance after accounting for patient identity. Within‐group analyses showed distinct temporal trajectories. In UC, the microbiota shifted towards donor and healthy controls: distance from donor decreased (Spearman's *ρ* = −0.68, *p* = 0.0025; *β* = −0.38, *p* = 0.001), RPCA Axis 1 moved towards controls (*ρ* = −0.57, *p* = 0.004), with a significant time × disease interaction indicating different trajectories between groups (*p* = 0.0047, power = 85.54%; Figure 2A–C), taxonomic log ratio increased (*β* = 1.40, *p* = 0.0001; *f* ^2^ = 1.45, power = 99.85%; Figure 3A–C), and functional log ratio shifted (*β* = 1.01, *p* = 6.5×10^−^^6^; *f* ^2^ = 2.42, power = 99.99%). In CD, no significant within‐group taxonomic changes were detected: distance from donor (*ρ* = −0.17, *p* = 0.40), Axis 1 (*ρ* = 0.23, *p* = 0.27; Figure 2A–C), and taxonomic log ratio (*β* = 0.10, *p* = 0.83; power = 5.00%; Figure 3A–C), with only a weak functional log‐ratio shift (*β* = 0.73, *p* = 0.0096; power = 36.51%). Post‐hoc power analyses showed that time × disease interactions for distance from donor and colonisation were underpowered (10.85% and 13.40%); their non‐significant results are inconclusive. The RPCA Axis 1 interaction (power = 85.54%) is reported as exploratory.

**FIGURE 2:**
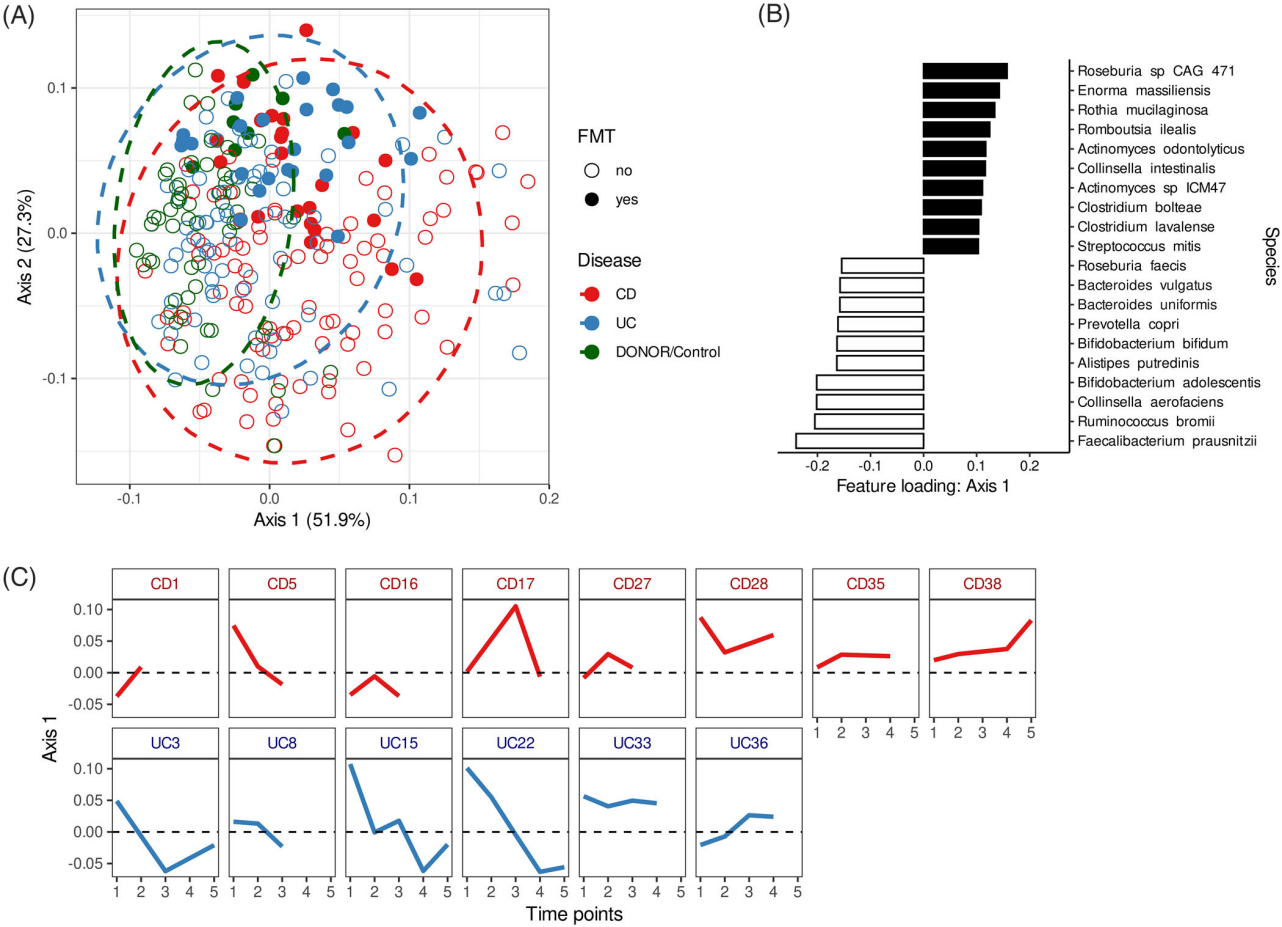
Changes in the taxonomic composition of stool metagenomes from inflammatory bowel disease (IBD) patients after faecal microbiota transplantation (FMT), relative to donors, external IBD patients, and healthy controls. (A) Robust Aitchison PCA (RPCA) biplot based on species‐level taxonomic profiles of patient metagenomes and external metagenomes from IBD patients and healthy subjects. Filled circles represent IBD patients who received FMT; open circles represent external IBD patients and healthy subjects. Colours denote disease groups. Aitchison distance was used as the dissimilarity metric, and 95% confidence ellipses around group centroids are shown. (B) Microbial feature rankings along Axis 1. The x‐axis represents feature loadings on Axis 1, with positive and negative values indicating taxa associated with the respective ends of the axis. (C) Temporal changes in IBD patients' stool metagenomic profiles along Axis 1. A decrease in Axis 1 values indicates a shift towards the healthy control region.

**FIGURE 3:**
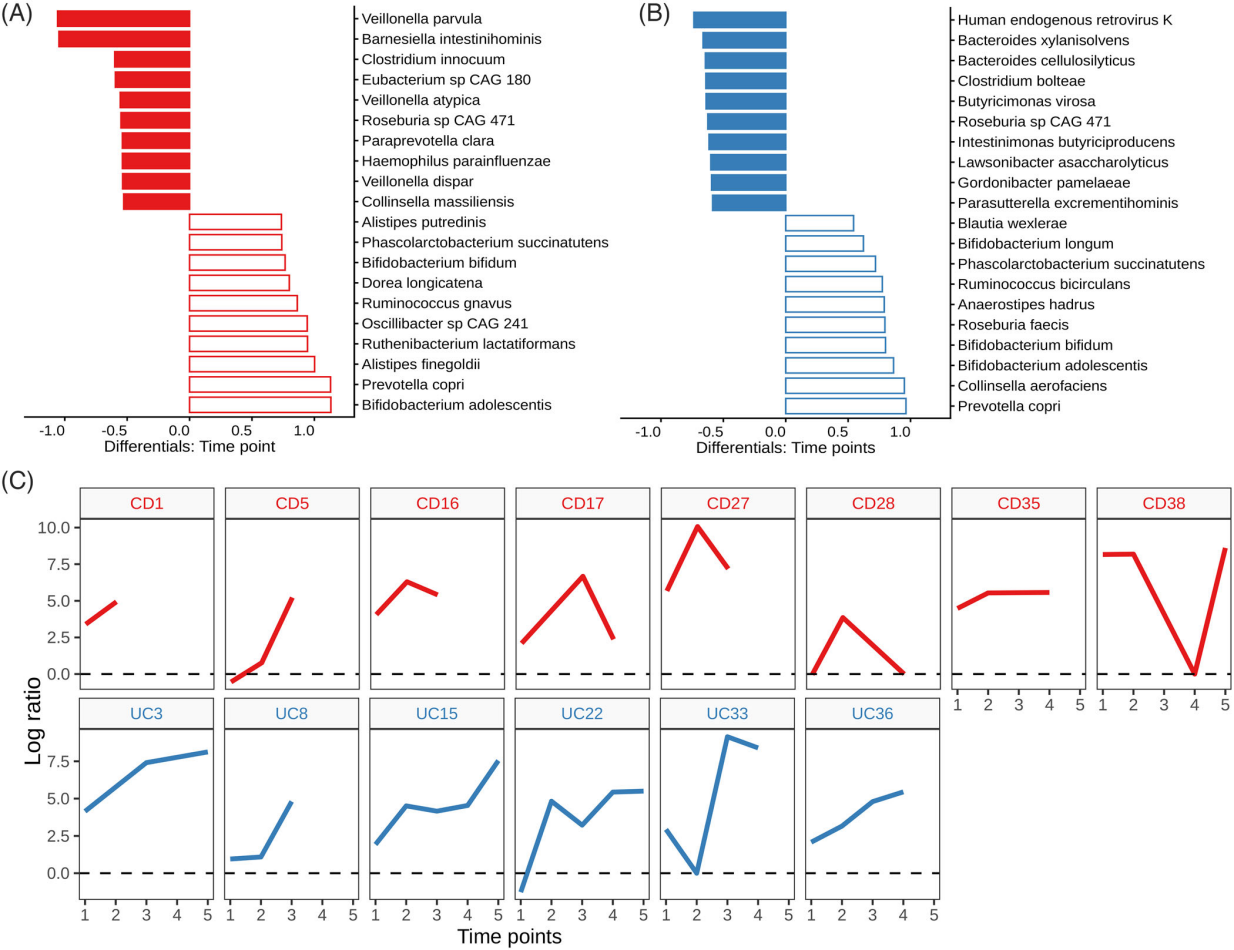
Changes in the taxonomic composition of patients' stool metagenomes over time. (A, B) Songbird taxonomy differential analysis shown as rank plots. The y‐axis shows microbial species, and the x‐axis shows differentials, representing the log‐fold change in features over time. Songbird values for the Crohn's disease (CD) group are shown in red, and those for the ulcerative colitis (UC) group are shown in blue. (C) Qurro sample plots showing feature log‐ratios in the context of the feature rankings presented in panels A and B.

These data provide preliminary evidence that, following FMT plus standard care, within‐group microbiota restructuring towards health was detectable in UC, with a coordinated shift towards donor and healthy‐control profiles; the improvement in taxonomic and functional content in UC is consistent with previous findings.^4^ In CD, no significant taxonomic restructuring was detected despite comparable clinical improvement; however, the analysis was underpowered, and the absence of a detected shift should not be interpreted as evidence of absence. The weak signal in CD may partly reflect differences in pathogenesis, including transmural inflammation, fibrostenotic changes, and CD‐specific host genetic factors.^5^ It may also indicate that clinical improvement in this group is driven to a greater extent by concurrent anti‐inflammatory treatment rather than by microbiota modulation, suggesting a potential limitation of FMT as a microbiota‐directed intervention in CD, as partly supported by previous studies.^6^ Antimicrobial peptide deficits and misfolding of α‐defensins arising from Paneth cell dysfunction may account for the lack of detectable microbiota restructuring in the CD group.^7^, ^8^ A weakened barrier may favour pathogens, whereas the indigenous microbiota, often resistant to these peptides, normally gains a competitive advantage over peptide‐sensitive organisms.^9^, ^10^ When peptides are deficient or misfolded, this advantage is lost, and the indigenous microbiota may be outcompeted. These interpretations remain exploratory and require confirmation.

The interpretative scope is constrained by several methodological limitations. The limited sample size restricted statistical power, particularly for interaction analyses, while the absence of a control arm receiving anti‐inflammatory treatment alone precluded attribution of the observed microbial alterations specifically to FMT. Demographic imbalances and the single‐centre design within a Russian cohort limit generalisability; accordingly, this investigation should be regarded as exploratory and proof‐of‐concept in nature. The coherent restructuring of taxonomic and functional profiles observed in UC lends support to the rationale for FMT as an adjunctive therapeutic strategy aimed at restoring gut microbiome composition and function. In CD, the absence of any detectable taxonomic response despite comparable clinical improvement, coupled with only a weak functional shift and no established difference in colonisation, warrants further investigation of alternative or combinatorial approaches, including repeated FMT, dietary intervention, or synthetic consortia supplementation. Should these observations be corroborated, they would suggest that IBD subtype‐specific considerations may be important in designing FMT protocols, and larger studies with longer follow‐up are warranted.

## AUTHOR CONTRIBUTIONS

Evgenii I. Olekhnovich—general data analysis strategy; experimental data analysis and visualisation; interpretation of obtained results; wrote the manuscript. Tatyana N. Kalachnyuk, Roman I. Matorin—management of CD and UC clinical cases; contribution to the design of the FMT clinical research protocol; performing FMT clinical procedure; contribution to the interpretation of obtained results. Dmitry E. Fedorov, Artem B. Ivanov—development and support of the bioinformatics computational protocols; data quality control management; the exploratory analysis of obtained metagenomes. Ksenia M. Klimina—supervising DNA isolation and shotgun sequencing. Vladimir A. Veselovsky, Daria I. Boldyreva and Andrey K. Larin—performed DNA isolation and shotgun sequencing. Svetlana V. Lyamina, Dmitriy N. Andreev, Igor V. Maev and Irina Balazs—support of the clinical data interpretation and management; contribution to the manuscript preparation. Elena S. Zhgun—donors’ and recipients’ clinical data management. Alexander V. Pavlenko—the design of the FMT clinical research protocol; management of donors' and recipients’ clinical data; supervising the collection of the stool biobank; supervising the FMT treatment procedure. Vadim M. Govorun—research idea; development of a general research concept; contribution to supervising the FMT project. Julia A. Bespyatykh—supervising the FMT project and contribution to the manuscript preparation. Elena N. Ilina—development of a general research concept; supervising the FMT project; supervising the design of the FMT clinical research protocol; contribution to the interpretation of obtained results; contribution to the manuscript preparation.

## CONFLICT OF INTEREST STATEMENT

The authors declare no conflict of interest.

## FUNDING INFORMATION

This project was partly supported by the Russian Science Foundation (grant No. 26‐15‐00478).

## ETHICS STATEMENT

This study was approved by the ethical committee of the Federal Research and Clinical Centre of Physical and Chemical Medicine (Protocol No. 2017/02, Apr 13, 2017).

## PATIENT CONSENT STATEMENT

Prior to the commencement of the study, donors and patients provided written informed consent.

## CLINICAL TRIAL REGISTRATION

Stool samples were collected from Russian patients who were over 18 years of age and diagnosed with Crohn's disease or ulcerative colitis as part of the clinical study NCT04011943 [https://www.clinicaltrials.gov/ct2/show/NCT04011943] to evaluate the effectiveness of faecal microbiota transplantation in improving health across a range of disorders.

## Supporting information



Supporting Information

Supporting Information

Supporting Information

Supporting Information

Supporting Information

Supporting Information

Supporting Information

## Data Availability

The raw metagenomic reads supporting the conclusions of this article are available in the NCBI repository under project ID PRJNA763503. All additional data and intermediate metagenomic analysis results were attached to the article as Supporting Information materials. The complete analysis, including code and results, is available as a Quarto document at https://github.com/JeniaOle13/FMT_IBD_project. The ASSNAKE pipeline, used for processing and generating taxonomic and functional profiles, is available at https://github.com/ASSNAKE. RECAST, used for identifying donor‐derived microbial diversity, is available at https://github.com/ctlab/recast.
