# Supplementary material for: Dynamic alterations in stool microbiota composition following faecal transplantation in patients with inflammatory bowel disease
Source: Clin Transl Med. 2026 Nov 5;16(11):e70888. doi: 10.1002/ctm2.70888 (PMC13736784; doi:10.1002/ctm2.70888)
Supplement: Supplementary file 1 [file CTM2-16-e70888-s001.docx]

**Study design**

This exploratory, single-arm, uncontrolled longitudinal case series enrolled 14 patients (10 males and 4 females; mean age 37.1 ± 13.7 years) with ileal (n = 7) or ileocolonic (n = 1) Crohn's disease (CD), or ulcerative colitis (UC) (n = 6). Patients were recruited at the Department of Gastroenterology of the Federal Research and Clinical Center of Physical-Chemical Medicine, Federal Medical and Biological Agency of Russia (Moscow, Russian Federation), between July 2017 and September 2019. Disease severity was assessed using the Harvey–Bradshaw Index (HBI) for CD [Harvey and Bradshaw, 1980] and the Simple Clinical Colitis Activity Index (SCCAI) for UC [Walmsley et al., 1998]. Inclusion criteria were as follows: a diagnosis of CD or UC established according to clinical symptoms, endoscopic findings, and histological criteria, in line with the current ECCO-ESGAR guidelines [Maaser et al., 2019; Sturm et al., 2019]. Six patients (5 with CD, 1 with UC) were newly diagnosed. During hospitalization, all patients received standard anti-inflammatory therapy, consisting of either 5-aminosalicylic acid (5-ASA) monotherapy or a combination of 5-ASA and glucocorticoids, with doses tailored to disease severity. Written informed consent was obtained from all participants prior to enrolment. Exclusion criteria were: age below 18 years; presence of other severe diseases, including other intestinal disorders (e.g., *Clostridioides difficile* infection), malignant neoplasms, cardiopulmonary failure, or serious hepatic and renal disease; and a follow-up period shorter than 3 months. The study was approved by the Ethics Committee of the Federal Research and Clinical Center of Physical-Chemical Medicine, Federal Medical and Biological Agency of Russia (Protocol no. 2017/02, April 13, 2017), and registered at ClinicalTrials.gov (NCT04011943). The study was conducted in accordance with the Declaration of Helsinki and Good Clinical Practice guidelines.

**Donor screening**

Healthy individuals (two males and four females; mean age 22.0 ± 3.6 years) were selected as fecal donors. All donors followed a standard well-balanced European diet throughout the study period and underwent clinical evaluation according to the protocol recommended by the European Consensus Conference on Fecal Microbiota Transplantation in Clinical Practice [Cammarota et al., 2017]. Donors were excluded if they had: (1) a family history of IBD or cancer; (2) infectious diseases; (3) metabolic syndrome; (4) arterial hypertension; (5) obesity; (6) diabetes mellitus; (7) use of antibacterial therapy within the six months prior to the study; or (8) a tattoo obtained within the six months prior to the study. All selected donors were in good health prior to stool donation and underwent routine medical checkups. They reported no dyspepsia or stool abnormalities within the year prior to donation. Routine clinical and laboratory examinations, including full blood count, blood biochemistry, lymphocyte subpopulation analysis, and urine analysis (physical, chemical, and microscopic examination of sediment), showed no abnormalities. Donor blood was additionally tested for: (1) syphilis; (2) hepatitis B and C; and (3) parasites, including protozoa and helminths. Donor stool underwent comprehensive examination: (1) testing for opportunistic microbial species; (2) testing for antibiotic-resistant bacteria, including methicillin-resistant *Staphylococcus aureus* (MRSA), vancomycin-resistant enterococci (VRE), extended-spectrum beta-lactamase (ESBL)- and carbapenemase-producing *Klebsiella pneumoniae* and *Escherichia coli*; (3) assessment of fecal calprotectin levels; (4) examination for *Clostridioides difficile* toxins A and B; (5) PCR testing for enteropathogenic viruses, including cytomegalovirus (CMV), Epstein-Barr virus, herpes simplex virus types 1 and 2 (HSV-1, HSV-2), human herpesvirus 6 (HHV-6), human adenoviruses (types B, C, E), noroviruses (types 1, 2), and rotaviruses (types A, B, C); and (6) testing for parasites, including protozoa and helminths. All laboratory tests revealed no abnormalities.

**Stool sampling and FMT procedure**

Donors received 100 ml of a magnesium sulfate solution, and stool was collected within 1–1.5 hours afterwards. Immediately after defecation, 150 ml of saline solution (0.9%) was added to the donor sample for every 30 g of feces and mixed to achieve a uniform consistency. If solid undigested particles were present, the suspension was filtered through a paper filter. Glycerin was added to a final concentration of 10%. The final suspension was transferred into a plastic container and frozen at −80 °C. All manipulations were completed within 90 minutes after donor defecation. The fecal suspension was used within 16 weeks from the date of freezing. Prior to the FMT procedure, the suspension was thawed at room temperature for 4–5 hours, or in a water bath at +37 °C one hour before the procedure. A total of 100 ml of the final donor fecal suspension was infused into the jejunum using an endoscope. Carbon dioxide insufflation was used to expand the jejunum and create an anaerobic environment. The donor fecal suspension was drawn into a syringe, and the syringe nozzle was inserted into the endoscope instrumental channel and slowly injected. After the procedure, the endoscope was removed from the patient's jejunum, along with the excess carbon dioxide. Following endoscope removal, the patient was placed in Fowler's position and asked to gently massage the abdomen while slowly turning from side to side for 30 minutes. These movements were intended to aid the distribution of the donor fecal suspension within the patient's gut [Scherbakov et al., 2019]. The FMT procedure was performed in parallel with the basic therapy. Following FMT, stool samples were collected from recipients into sterile plastic containers at five time points: (1) before FMT; (2) 2 days after FMT; (3) 7 days after FMT; (4) 14 days after FMT; and (5) 30 days after FMT. In total, 48 stool samples were collected from patients (24 from CD and 24 from UC) and eight stool samples were collected from donors. Thus, 48 of 70 expected patient samples were available (overall completeness 68.6%), with missing samples more frequent at days 14 and 30.

**DNA isolation and shotgun sequencing of stool samples**

DNA was isolated from stool samples using a MagNa Pure Compact Nucleic Acid Isolation Kit I (Roche, Basel, Switzerland) according to the manufacturer's instructions. Extracted DNA was eluted in 50 µl of the supplied buffer and stored at −18 °C. For shotgun metagenomic library preparation, 250 ng of genomic DNA was used. DNA was sonicated using a Covaris S220 System (Covaris, Woburn, MA, USA), and the fragment size (400–500 bp) and quality were assessed with an Agilent 2100 Bioanalyzer (Agilent, Santa Clara, CA, USA). Paired-end libraries were prepared using the NEBNext Ultra II DNA Library Prep Kit (New England Biolabs, Ipswich, MA, USA) and indexed with NEBNext Multiplex Oligos for Illumina (96 Index Primers; New England Biolabs, Ipswich, MA, USA). Libraries were quantified using the Quant-iT DNA Assay Kit, High Sensitivity (Thermo Scientific, Waltham, MA, USA). Sequencing was performed on a HiSeq 2500 platform (Illumina, San Diego, CA, USA) with 2 × 125 bp reads.

**Data analysis and visualization**

*Metagenomic data processing*

The raw metagenomic data were quality assessed using FastQC (v0.11.9) [<https://github.com/s-andrews/FastQC>]. Technical sequences and low-quality bases were trimmed using Trimmomatic v0.38 [Bolger et al., 2014] with the following parameters: ILLUMINACLIP:TruSeq3-PE-2.fa:2:3:10, SLIDINGWINDOW:4:15, LEADING:25, TRAILING:25, and MINLEN:50. Human sequences were removed by mapping reads against the human genome GRCh38 (hg38) using bbmap v38.90 [Bushnell, 2014], and only non-human reads were retained for downstream analysis. Metagenomic taxonomic profiling was performed using MetaPhlAn3 v3.0.0 with the CHOCOPhlAn v201901 database [Beghini et al., 2021] with default parameters. For functional analysis, HUMAnN2 v2.8.1 [Franzosa et al., 2018] was run with the KEGG database (release 2020-03-09) [Kanehisa et al., 2017] to generate KO-level functional profiles. The described computational steps were implemented in the Assnake metagenomics pipeline [<https://github.com/ASSNAKE>].

*Alpha and beta diversity analysis*

Species-level relative abundances were filtered to remove rare and low-prevalence taxa (present in fewer than 20% of samples) prior to downstream analysis. Alpha diversity was assessed using the Shannon index, and beta diversity was computed as the Aitchison distance between centred log-ratio (CLR) transformed species abundances [Aitchison, 1992; Aitchison, 1997] using Juan José Egozcue `mini_composition` custom library. Sample ordination was performed by non-metric multidimensional scaling (NMDS) based on the Aitchison distance. To verify that group comparisons were not confounded by unequal multivariate dispersion, Permutational Analysis of Multivariate Dispersions (PERMDISP) was performed. Within-subject effects (time point, clinical index) were tested with Permutational Multivariate Analysis of Variance (PERMANOVA) using patient stratification (strata = patient id) to account for repeated measures. All three analyses (NMDS, PERMDISP, and PERMANOVA) were implemented using the vegan R package (v2.7-6) [Oksanen et al., 2026]. Between-subject effects (disease, donor) were tested using Multiple Regression on distance Matrices (MRM) with binary indicators of shared disease, donor, and patient identity, implemented in the ecodist R package (v2.1.3) [Goslee and Urban, 2007].

*Trajectory analysis using DEICODE and Songbird*

To place the FMT cohort in the context of an independent IBD reference dataset, robust Aitchison PCA was performed using DEICODE (v0.2.3) [Martino et al., 2019], implemented in the QIIME 2 framework [Bolyen et al., 2019], on a merged dataset combining our samples with 218 previously published metagenomes from IBD patients and healthy subjects [Franzosa et al., 2018]; the resulting ordination provided a shared space to formally compare temporal trajectories between CD and UC, and the Axis 1 coordinates were extracted for each sample and used as a quantitative phenotype in downstream statistical models. Differential ranking of taxa and KEGG Orthology (KO) features by their association with time was performed using Songbird (v1.0.3) [Morton et al., 2019], implemented in the QIIME 2 framework [Bolyen et al., 2019], with the multinomial regression model fitted with time point as the primary covariate within each disease group separately, yielding regression coefficients that quantify the direction and magnitude of each feature's association with time; because the two disease groups were modelled independently, the resulting rankings for CD and UC are based on distinct feature sets and are not directly comparable on a common scale, and a combined time × disease interaction model was therefore not fitted. Sample-level log ratios were extracted using Qurro [Fedarko et al., 2020], also implemented in the QIIME2 framework, by selecting features with an absolute Songbird regression coefficient greater than 0.3 as numerator and denominator sets and computing the log ratio of these sets per sample; the resulting log ratios were used as quantitative phenotypes in downstream statistical models. Gene set enrichment analysis (GSEA) was then performed on KO-level data using the piano Bioconductor package (v2.28.0) [Väremo et al., 2013] with the "median" algorithm and Benjamini–Hochberg correction, with a gene set significance threshold of adjusted p < 0.05, using the full ranked KO lists to provide functional interpretation of the results.

*Donor-derived species identification with RECAST*

The RECAST (Recipient intestinE Colonisation AnalysiS Tool) approach (v1.0.0) was used to detect donor-derived microbial species in the post-FMT recipient metagenomes. Validation of RECAST, including benchmarking on simulated metagenomes and a control set of biologically unrelated metagenomes, is described in the original publication [Olekhnovich et al., 2021]. RECAST classifies metagenomic reads by comparing three samples (the donor, the pre-FMT recipient, and the post-FMT recipient) using de Bruijn graph-based coverage criteria. Reads are assigned to eight categories according to their origin and persistence after transplantation: two categories describe reads from the donor, two describe reads from the pre-FMT recipient, and four describe reads from the post-FMT recipient based on whether they match the donor, the baseline, both, or neither. A minimum coverage breadth of 90% was applied for reads to be assigned to the "found" class. Reads detected in both the donor and the post-FMT recipient that were not classified as originating from the baseline or from an unknown source were taxonomically classified using MetaPhlAn3 (v3.0.0) with the CHOCOPhlAn v201901 database [Beghini et al., 2021]. The number of colonising species per post-FMT sample (abundance threshold of 100 reads) was summarised and analysed with the same statistical models described in the following section.

*Statistical modeling and power analysis*

Aitchison distances from baseline and from the assigned donor were computed for each post-FMT sample, and DEICODE Axis 1 coordinates, Songbird log ratios for taxa and KO features, and the number of donor-derived species colonising the recipient gut were used as additional response variables. Patient ID was included as a random intercept in the main longitudinal models where the random-effect variance was non-negligible; time point and donor identity were included as fixed effects. Where the random-effect variance was negligible (as indicated by likelihood ratio tests), generalized linear models without random effects were fitted for comparison and used for the interaction analyses. Linear mixed models were fitted using lme4 (v2.0.6) [Bates et al., 2015] and lmerTest (v3.2.1) [Kuznetsova et al., 2017]. To test whether temporal trajectories differed between disease groups, models including a time point × disease interaction were fitted and compared with the corresponding main-effects models by likelihood ratio test, with estimated marginal trends of time point per disease group obtained using emmeans (v2.0.4) [Lenth, 2024]. For the interaction model on RPCA Axis 1, age and gender were additionally included as fixed covariates. Explained variance was quantified with marginal and conditional R² using performance (v0.18.1) [Lüdecke et al., 2021], standardised coefficients were obtained with effectsize (v1.0.3) [Ben-Shachar et al., 2020], and Cohen's f² was computed for key predictors as the increment in R² relative to a reduced model without the predictor of interest. Post-hoc power analysis was used as an additional filter to distinguish adequately supported findings from those limited by insufficient sensitivity. Power for fixed-effects models was computed using pwr (v1.3.0) [Champely, 2020], and simulation-based power for mixed-effects models using simr (v1.0.11) [Green and MacLeod, 2016]; the choice of method depended on whether the random-effect variance was non-negligible. Minimum detectable effect sizes were computed at 80% power.

*Data manipulation, visualisation, and reproducibility*

Data manipulation and visualisation were performed using dplyr (v1.2.1) [Wickham et al., 2025], tidyr (v1.3.2) [Wickham et al., 2026], reshape2 (v1.4.5) [Wickham, 2007], stringr (v1.6.0) [Wickham, 2026], ggplot2 (v4.0.3) [Wickham, 2016], pheatmap (v1.0.13) [Kolde, 2019], RColorBrewer (v1.1.3) [Neuwirth, 2022], DT (v0.34.0) [Xie et al., 2026], and downloadthis (v0.5.0) [Fellows, 2025]. All analyses were conducted in R (v4.6.1) [R Core Team, 2026] and reported using Quarto (v1.9.36) [Allaire et al., 2026].

**References**

1. Harvey RF, Bradshaw JM. A simple index of Crohn's-disease activity. *The Lancet*. 1980;315(8167):514.

2. Walmsley RS, Ayres RCS, Pounder RE, Allan RN. A simple clinical colitis activity index. *Gut*. 1998;43(1):29–32.

3. Maaser C, Sturm A, Vavricka SR, et al. ECCO-ESGAR Guideline for Diagnostic Assessment in IBD Part 1: Initial diagnosis, monitoring of known IBD, detection of complications. *Journal of Crohn's and Colitis*. 2019;13(2):144–164.

4. Sturm A, Maaser C, Calabrese E, et al. ECCO-ESGAR Guideline for Diagnostic Assessment in IBD Part 2: IBD scores and general principles and technical aspects. *Journal of Crohn's and Colitis*. 2019;13(3):273–284.

5. Cammarota G, Ianiro G, Tilg H, et al. European consensus conference on faecal microbiota transplantation in clinical practice. *Gut*. 2017;66(4):569–580.

6. Scherbakov PL, Belova ND, Generozov EV, et al. Faecal Transplant in GIT Treatment (Pilot Clinical Experience). *DoctorRu*. 2019;158(3):40–46.

7. Bolger AM, Lohse M, Usadel B. Trimmomatic: a flexible trimmer for Illumina sequence data. *Bioinformatics*. 2014;30(15):2114–2120.

8. Bushnell B. BBMap: A Fast, Accurate, Splice-Aware Aligner. Lawrence Berkeley National Laboratory. LBNL Report #: LBNL-7065E. 2014.

9. Beghini F, McIver LJ, Blanco-Míguez A, et al. Integrating taxonomic, functional, and strain-level profiling of diverse microbial communities with bioBakery 3. *eLife*. 2021;10:e65088.

10. Franzosa EA, McIver LJ, Rahnavard G, et al. Species-level functional profiling of metagenomes and metatranscriptomes. *Nature Methods*. 2018;15(11):962–968.

11. Kanehisa M, Furumichi M, Tanabe M, Sato Y, Morishima K. KEGG: new perspectives on genomes, pathways, diseases and drugs. *Nucleic Acids Research*. 2017;45(D1):D353–D361.

12. Aitchison J. On criteria for measures of compositional difference. *Mathematical Geology*. 1992;24(4):365–379.

13. Aitchison J. The one-hour course in compositional data analysis or compositional data analysis is simple. In: Pawlowsky-Glahn V, editor. Proceedings of the III Annual Conference of the International Association for Mathematical Geology (vol. I). Barcelona, Spain: CIMNE; 1997. p. 3–35.

14. Oksanen J, Simpson GL, Blanchet FG, et al. vegan: Community Ecology Package (Version 2.7-6) [Computer software]. 2026.

15. Goslee SC, Urban DL. The ecodist package for dissimilarity-based analysis of ecological data. *Journal of Statistical Software*. 2007;22(7):1–19.

16. Bates D, Mächler M, Bolker B, Walker S. Fitting Linear Mixed-Effects Models Using lme4. *Journal of Statistical Software*. 2015;67(1):1–48.

17. Kuznetsova A, Brockhoff PB, Christensen RHB. lmerTest package: Tests in linear mixed effects models. *Journal of Statistical Software*. 2017;82(13):1–26.

18. Lenth R. emmeans: Estimated Marginal Means, aka Least-Squares Means. R package version 2.0.4. 2024.

19. Lüdecke D, Ben-Shachar MS, Patil I, Waggoner P, Makowski D. performance: An R Package for Assessment, Comparison and Testing of Statistical Models. *Journal of Open Source Software*. 2021;6(60):3139.

20. Ben-Shachar MS, Lüdecke D, Makowski D. effectsize: Estimation of Effect Size Indices and Standardized Parameters. *Journal of Open Source Software*. 2020;5(56):2815.

21. Martino C, Morton JT, Marotz CA, et al. A Novel Sparse Compositional Technique Reveals Microbial Perturbations. *mSystems*. 2019;4(1).

22. Franzosa EA, Sirota-Madi A, Avila-Pacheco J, et al. Gut microbiome structure and metabolic activity in inflammatory bowel disease. *Nature Microbiology*. 2018;4(2):293–305.

23. Morton JT, Marotz C, Washburne A, et al. Establishing microbial composition measurement standards with reference frames. *Nature Communications*. 2019;10(1).

24. Fedarko MW, Martino C, Morton JT, et al. Visualizing 'omic feature rankings and log-ratios using Qurro. *NAR Genomics and Bioinformatics*. 2020;2(2).

25. Bolyen E, Rideout JR, Dillon MR, et al. Reproducible, interactive, scalable and extensible microbiome data science using QIIME 2. *Nature Biotechnology*. 2019;37(8):852–857.

26. Väremo L, Nielsen J, Nookaew I. Enriching the gene set analysis of genome-wide data by incorporating directionality of gene expression and combining statistical hypotheses and methods. *Nucleic Acids Research*. 2013;41(8):4378–4391.

27. Olekhnovich EI, Ivanov AB, Ulyantsev VI, Ilina EN. Separation of Donor and Recipient Microbial Diversity Allows Determination of Taxonomic and Functional Features of Gut Microbiota Restructuring following Fecal Transplantation. *mSystems*. 2021;6(4).

28. Champely S. pwr: Basic Functions for Power Analysis. R package version 1.3-0. 2020.

29. Green P, MacLeod CJ. simr: An R package for power analysis of generalised linear mixed models by simulation. *Methods in Ecology and Evolution*. 2016;7(4):493–498.

30. Wickham H, François R, Bryan J, Müller K. dplyr: A Grammar of Data Manipulation. R package version 1.2.1. 2025.

31. Wickham H, Vaughan D, Girlich M. tidyr: Tidy Messy Data. R package version 1.3.2. 2026.

32. Wickham H. Reshaping data with the reshape package. *Journal of Statistical Software*. 2007;21(12):1–20.

33. Wickham H. stringr: Simple, Consistent Wrappers for Common String Operations. R package version 1.6.0. 2026.

34. Wickham H. ggplot2: Elegant Graphics for Data Analysis. Springer-Verlag New York. 2016.

35. Kolde R. pheatmap: Pretty Heatmaps. R package version 1.0.13. 2019.

36. Neuwirth E. RColorBrewer: ColorBrewer Palettes. R package version 1.1-3. 2022.

37. Xie J, Cheng J, Tan X. DT: A Wrapper of the JavaScript Library 'DataTables'. R package version 0.34.0. 2026.

38. Fellows FM. downloadthis: Download Buttons for R Markdown with 'htmltools'. R package version 0.5.0. 2025.

39. R Core Team. R: A Language and Environment for Statistical Computing. R Foundation for Statistical Computing, Vienna, Austria. 2026.

40. Allaire JJ, Teague C, Scheidegger C, Xie Y, Dervieux C. Quarto. Software. R package version 1.9.36. 2026.
